# Fenofibrate Differently Affects the Heart’s Morphology and Metabolism in Young and Old Rats

**DOI:** 10.3390/ijms26168038

**Published:** 2025-08-20

**Authors:** Agata Wrońska, Jacek Kieżun, Zbigniew Kmieć

**Affiliations:** 1 Department of Histology, Faculty of Medicine, Medical University of Gdańsk, 80-210 Gdańsk, Poland; 2 Department of Anatomy and Histology, School of Medicine, University of Warmia and Mazury in Olsztyn, 10-082 Olsztyn, Poland; jacek.kiezun@uwm.edu.pl (J.K.); zbigniew.kmiec@uwm.edu.pl (Z.K.)

**Keywords:** aging, fenofibrate, heart

## Abstract

Fenofibrate (FF), a lipid-lowering drug, may decrease the risk of cardiovascular diseases in some pathological settings, yet data on its cardiac effects in physiological aging is scarce. To determine FF and age effects on the heart’s morphology and expression of metabolism-related genes, we treated young and old male rats for 30 days with 0.1% or 0.5% FF. FF did not affect serum activities of LDH and creatine kinase in both age groups. Upon FF treatment the structure of the heart muscle did not change in young rats; however, 0.5% FF increased the abundance of collagen fibers in old rats, and lipid accumulation in cardiomyocytes in young and old animals. FF increased immunoreactivity of the hypertrophy marker NPPA that was more pronounced in old than in young rats, while VEGFB immunoreactivity did not change. FF upregulated phospho-AMPK and PGC1α protein levels only in the cardiac muscle of old rats, while in both age groups it mildly increased the expression of selected fatty acid oxidation genes. We conclude that the cardiac muscle response to FF is dose-dependent and influenced by age. The observed negative impact of high-dose FF in the hearts of aged rats underscores the importance of dose optimization in the elderly.

## 1. Introduction

The aging of the global population contributes to cardiovascular diseases (CVDs) remaining the leading cause of morbidity and mortality worldwide, despite improvements in medical care [1,2]. Besides older age itself, hypertension, hyperlipidemia, obesity, and diabetes are the key risk factors for CVDs, including atherosclerosis, myocardial infarction, and stroke [3,4,5]. Consequently, lipid-lowering therapeutics may decrease CVD risk. Among such drugs, fenofibrate (FF) is indicated for the treatment of mixed type dyslipidemia and hypertriglyceridemia, especially in patients with atherogenic dyslipidemia or with statin intolerance [6]. Several large-scale clinical trials demonstrated cardioprotective potential of FF in patients with type 2 diabetes mellitus (T2DM). In the Fenofibrate Intervention and Event Lowering in Diabetes (FIELD) study, FF showed a reduction in the progression of microvascular complications and a trend towards reduced non-fatal myocardial infarction events [7]. In the Action to Control Cardiovascular Risk in Diabetes (ACCORD) study, FF reduced the risk of major cardiovascular events only in patients with elevated triglycerides and low HDL cholesterol levels [8]. Moreover, in the ACCORD Lipid trial of patients with T2DM receiving simvastatin, FF reduced hospitalizations related to heart failure and cardiovascular death [9]. Furthermore, FF significantly decreased the composite of myocardial infarction, stroke, percutaneous coronary revascularization, and cardiac death in Korean patients with diabetes [10]. However, it is not known how FF affects the heart muscle biology in a non-pathological setting of physiological aging.

At the molecular level, FF primarily acts as an agonist of peroxisome proliferator-activated receptor alpha (PPARα), whose activation orchestrates a multifaceted molecular response [11], which may collectively contribute to a cardioprotective effect. PPARα drives the transcription of target genes that promote fatty acid oxidation (FAO), reduce triglyceride synthesis, and enhance HDL production [12]. PPARα activation may also counter the inflammatory process [13,14,15] and promote an antioxidant response [16,17].

There is a scarcity of data on the effects of FF as a function of age. Clinical guidelines often lack specific recommendations for older-aged individuals [18], even though the pharmacokinetics of multiple medications may differ in this population due to aging-associated changes in organs’ functions [19,20]. Bench studies are typically carried out in young-adult animals and rarely examine particular drugs in old organisms. However, in our previous studies in a rat model we observed clear age-related differences in the response to FF: FF-induced impairment of liver function and more pronounced changes in hepatocyte ultrastructure in old than in young rats [21]; increased UCP1 protein content and mRNA expression of targets involved in oxidative metabolism in the adipose tissue of predominantly young rats [22]; activation of cellular antioxidant defenses in the kidney of only young rats; and elevated fibrotic tissue area and pro-inflammatory genes’ expression after high-dose FF in old rats [23].

Aging of the heart is associated with morphological changes, including hypertrophy and fibrosis, which underlie the higher prevalence of clinical conditions such as left ventricular hypertrophy, atrial fibrillation, a decline in diastolic function, and an overall decline in exercise capacity [24]. At the molecular level, age-related changes in the cardiac transcriptome and cellular signaling pathways encompass some crucial targets of FF action, such as AMP-activated protein kinase (AMPK), PPAR-γ coactivator 1-α (PGC1α), and sirtuins (e.g., SIRT1, SIRT3). Therefore, insight into the molecular basis behind age-related differences in the cardiac muscle response to FF could provide ground for improvement of therapy in the elderly.

The aim of the present study was to compare the morphology and expression of important metabolism-related targets in the cardiac muscle of young and old rats that received two doses of FF in chow pellets for 30 days.

## 2. Results

### 2.1. Lipids and Biochemical Markers in Blood Serum

Old as compared to young control rats had elevated concentrations of triglycerides and total cholesterol in blood serum (Table 1). High-dose FF decreased triglycerides’ concentration only in old rats, while cholesterol levels decreased after low-dose FF in young rats and after high-dose FF in the old ones (Table 1).

The blood serum levels of lactate dehydrogenase (LDH) and creatine kinase (CK) serve as useful biomarkers of muscle tissue integrity. Neither aging nor FF treatment affected LDH and CK serum levels in our study (Table 1).

Serum creatinine levels were similar in young and old control rats (Table 1). High-dose FF increased serum creatinine levels in both age groups, but with a more pronounced effect in old animals (Table 1).

### 2.2. Fenofibrate Effects on the Morphology of Cardiac Muscle

**Figure 1:**
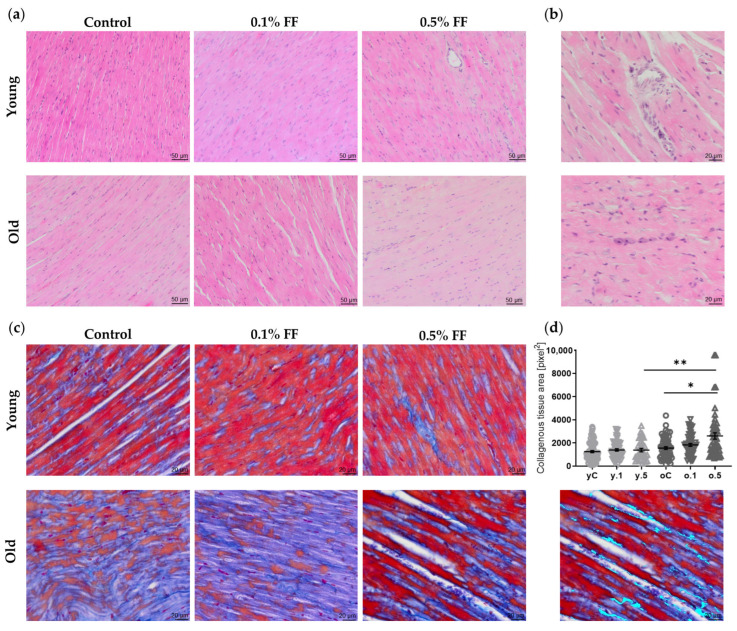
Histological staining of heart left ventricular sections from young and old rats treated with 0.1% or 0.5% fenofibrate (FF). (**a**,**b**) Representative photomicrographs of hematoxylin and eosin, and (**c**) Mallory’s trichrome staining. In panel (**a**), the unstained areas indicate interstitial tissue (extramyocyte space). Panel (**b**) shows cardiac tissue infiltration by mononuclear cells. Panel (**d**) shows quantification of collagen-rich areas, performed using neuronal network-assisted image analysis; an exemplary image is included. Scale bars: 50 µm in (**a**), 20 µm in others. Data are presented as mean ± SEM, based on ≥6 regions of interest (ROIs) per slide and 4–6 animals per group. * *p* < 0.05, ** *p* < 0.01. Abbreviations: yC, oC—young and old control rats; y.1, o.1, y.5, o.5—young and old rats treated with 0.1% or 0.5% FF, respectively.

The histological structure of rat myocardium within the wall of the left ventricle was examined in HE- and Mallory-stained sections (Figure 1). Young control rats showed neatly arranged, branching fibers, with cardiomyocytes’ nuclei located centrally (Figure 1a). Very small amounts of collagen were detected in the myocardial interstitial areas and were more abundant in perivascular areas (Figure 1b,c).

Treatment of young rats with 0.1% or 0.5% FF had no effect on the cardiac muscle morphology (Figure 1a). Mallory staining revealed no change in the abundance of collagenous tissue in FF-treated groups compared to young control rats (Figure 1c).

The myocardium of old as compared to young rats revealed a more disorganized arrangement, with widened spaces between fibers and some congested blood vessels (Figure 1a). Mallory staining revealed that the area of collagenous tissue in perivascular and interstitial regions was comparable to that observed in the young rats’ myocardium (Figure 1c).

Treatment of the old rats with FF at either dose did not affect the myocardial morphology in HE staining. Both 0.1% and 0.5% FF groups revealed moderately disorderly tissue architecture, with increased interstitial spaces between fibers and infiltrations of mononuclear cells in some regions (Figure 1b). Only the higher FF dose significantly increased collagen deposition in perivascular and interstitial areas (Figure 1c,d).

### 2.3. Impact of Fenofibrate on Lipid Droplets’ Area in Cardiomyocytes

**Figure 2:**
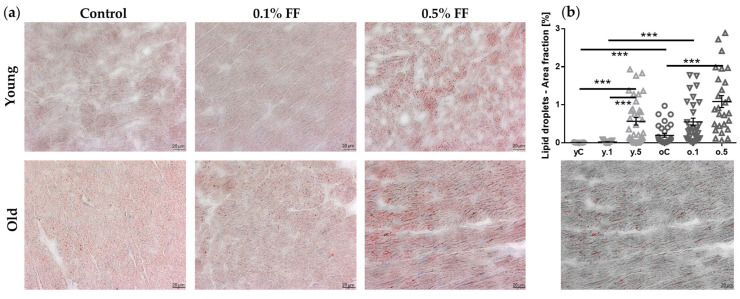
Histochemical analysis of lipid deposition in the heart tissue of young and old rats treated with 0.1% or 0.5% fenofibrate (FF). (**a**) Representative images of Oil Red O-stained cryosections; scale bars: 20 µm. (**b**) Quantification of lipid deposition, with an example of image analysis (red overlays on a greyscale background). Quantification was based on image analysis of at least six ROIs, from two slides per rat, with 4–6 rats per group. Data are presented as mean ± SEM, horizontal lines indicate group means. *** *p* < 0.001.

We performed Oil Red O staining to assess lipid deposition in the cardiac muscle cells. In cardiomyocytes of old as compared to young control rats, the lipid deposits were more abundant. In both age groups, low-dose FF did not significantly affect lipid accumulation, but high-dose FF increased the area fraction occupied by lipid droplets (Figure 2).

### 2.4. Fenofibrate Effects on Immunohistochemical Markers of Cardiac Hypertrophy

We performed immunohistochemical (IHC) analyses of markers associated with cardiac remodeling: NPPA (natriuretic peptide precursor A) and VEGFB (vascular endothelial growth factor B).

**Figure 3:**
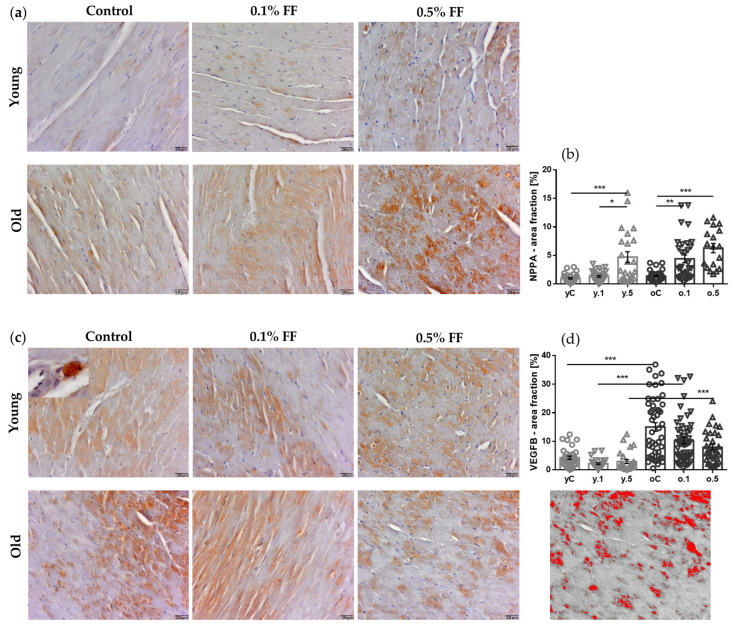
Immunohistochemical staining for markers of cardiac remodeling in the heart muscle of fenofibrate (FF)-treated and control rats. (**a**) Representative images of NPPA and (**c**) VEGFB staining; scale bar 20 μm; the 3× magnified inset shows a mononuclear cell in perivascular space. (**b**) Quantification of NPPA- and (**d**) VEGFB-positive area fractions, performed using image analysis across at least six ROIs, with 4–6 rats per group. Data are presented as mean ± SEM. * *p* < 0.05, ** *p* < 0.01, *** *p* < 0.001. The photograph below the graphs in (**d**) shows an example of image analysis.

NPPA immunoreactivity was observed in cardiomyocytes (Figure 3a), with similar levels in control young and old rats. Treatment of young rats with FF was associated with an increase in NPPA-positive area, with a significant difference between low and high doses. In old rats, both doses of FF caused more pronounced increases in the NPPA-stained area (Figure 3a,b).

VEGFB immunoreactivity was observed in cardiomyocytes and mononuclear cells in perivascular areas (Figure 3c, inset). VEGFB immunoreactivity levels were three-fold higher in the myocardium of control old than young rats. In both age groups, no significant changes were noted after treatment with FF, though there was a dose-related tendency for reduced immunoreactivity (Figure 3c,d).

### 2.5. Impact of Fenofibrate on the Expression of Genes and Proteins Involved in Cardiac Muscle Energy Metabolism

Since FF activates PPARα to stimulate cellular oxidative metabolism [25], we measured gene and protein expression of key regulators and effectors of the involved metabolic pathways.

**Figure 4:**
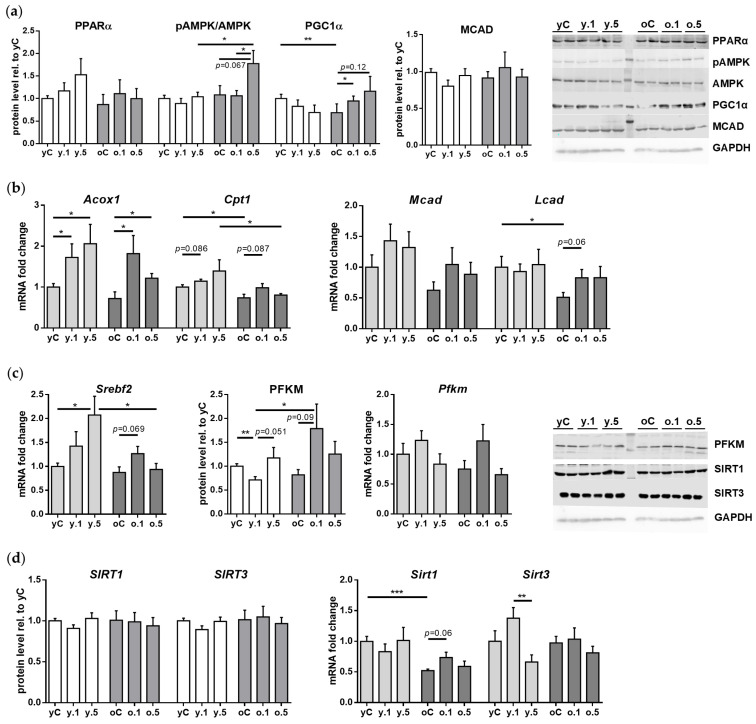
Effects of fenofibrate (FF) on the expression of genes and proteins related to cardiac energy metabolism in young and old rats. (**a**) Relative protein expression of PPARα, pAMPK/AMPK, PGC1α, and MCAD based on immunoblot quantification; representative blots are shown. (**b**) Relative mRNA expression of *Acox1*, *Cpt1*, *Mcad*, and *Lcad* (qPCR). (**c**) Relative mRNA levels of *Srebf2*, and protein and gene expression of PFKM. (**d**) Protein and gene expression of SIRT1 and SIRT3. Data in graphs represent mean ± SEM from 4–6 (WB) or 6–9 (qPCR) rats per group. * *p* < 0.05, ** *p* < 0.01, *** *p* < 0.001. Abbreviations: yC—young control rats; y.1, y.5—young rats receiving 0.1% or 0.5% FF, respectively; oC, o.1, o.5—corresponding groups of old rats.

The basic levels of PPARα were similar in the hearts of young and old control rats. Interestingly, treatment with either dose of FF did not affect the relative protein content of PPARα in the cardiac muscle of young or old rats (Figure 4a).

Likewise, young and old rats showed no age-related differences in the ratio of active phospho-AMPK (Thr172) to AMPK, a key cellular energy sensor. FF treatment did not affect this ratio in young rats; however, in the old ones 0.5% FF showed a tendency to increase the *p*-AMPK/AMPK ratio (Figure 4a).

PGC1α, a master regulator of mitochondrial energy metabolism [26], can be activated by PPARα. PGC1α protein level was two-fold lower in old than in young control rats. In the cardiac muscle of young rats, neither dose of FF altered the relative content of PGC1α. However, 0.1% FF increased PGC1α level in the cardiac muscle of old rats (Figure 4a).

The expression of genes encoding FAO enzymes may be induced through PPARα activation [25]. In the cardiac muscle of control old animals, the basic expression levels of *Cpt1* (carnitine palmitoyltransferase 1) and *Lcad* (acyl-CoA dehydrogenase, long chain) genes were lower than in the young ones, whereas the mRNA levels of *Acox* (acyl-CoA oxidase 1) and *Mcad* (acyl-CoA dehydrogenase, medium chain), as well as MCAD protein were similar in both age groups (Figure 4a,b). Interestingly, 0.1% FF and 0.5% FF in young rats increased the gene expression of *Acox1*, but not *Cpt1*. Moreover, *Mcad* gene and protein expression and *Lcad* mRNA levels showed no changes upon FF treatment in young rats (Figure 4a,b). In old rats, only the lower dose of FF increased *Acox1* expression, while neither dose of FF significantly affected the expression of *Cpt1*, *Mcad*, or *Lcad* genes (Figure 4b).

We measured the mRNA levels of sterol regulatory element-binding protein 2 gene (*Srebf2*), which—through regulation of genes for cholesterol biosynthesis and uptake—participates in lipid metabolism [27]. The mRNA expression of *Srebf2* in control rats was unaffected by aging. In young rats, the higher dose of FF increased *Srebf2* expression, while in the old ones neither dose significantly affected *Srebf2* mRNA levels (Figure 4c).

We also analyzed the expression of phosphofructokinase, muscle isoform (PFKM), which catalyzes the rate-limiting step of glycolysis and plays an important role in cardiovascular diseases [28]. The mRNA and protein PFKM levels were unaffected by aging. Treatment of young rats with 0.1% FF was associated with reduced PFKM protein expression, although without corresponding changes at the transcriptional level. However, no changes in PFKM level (relative to control) were observed after administration of 0.5% FF (Figure 4c). In contrast to the young, in old rats PFKM protein levels tended to increase after 0.1% FF, but not after the higher dose, whereas *Pfkm* mRNA expression was unaffected by the treatment (Figure 4c).

Lastly, the expression of two sirtuins involved in the regulation of energy metabolism, SIRT1 and SIRT3, did not change with age and was also mostly unaffected by treatment with FF. Neither age group of FF-treated rats showed any changes in SIRT1 and SIRT3 protein and mRNA levels in the heart, relative to control. Interestingly, only in young rats, *Sirt3* gene expression was lower after administration of 0.5% FF as compared to 0.1% FF (Figure 4d).

## 3. Discussion

Fenofibrate, a PPARα agonist, is primarily applied to reduce serum triglycerides and increase high-density lipoprotein cholesterol (HDL-C) levels [29]. Recently, FF pleiotropic effects in non-hepatic tissues have gained the attention of clinicians and the scientific community. However, there are scarce data on FF effects in the context of aging. Given that aging-associated alterations in the cardiac muscle may contribute to increasing prevalence of cardiovascular diseases in the elderly [4,5], we examined how FF affects the heart morphology and key metabolic targets in old as compared to young rats. Our results show that while low-dose FF may confer some mild benefits in the aged heart, the five-times higher dose may lead to adverse remodeling, reflected by increased collagen deposition and lipid droplet accumulation.

In our experimental model, we examined the effects of FF in the heart under non-pathological aging conditions. As commonly observed [30,31], old rats were characterized by elevated lipid levels in blood serum. In consequence, the lipid-lowering action of FF was more pronounced in old than in young rats, as reported in our previous work [21]. We also checked that the blood serum levels of muscle tissue integrity biomarkers were not affected by either aging or treatment with FF. Interestingly, in young-adult rats administered very high doses of FF or a selective PPARα agonist CP-778875 for forty days, increases in serum troponin I indicated cardiac muscle damage, even though serum creatine kinase activity remained unchanged [32]. In humans, while myopathy has been occasionally associated with FF therapy in patients with renal impairment or polypharmacy [33], we have found no reports of cardiomyopathy. In our study, treatment with high-dose FF increased serum creatinine levels, particularly in old rats [23]. Although such elevation might suggest renal impairment, in patients it appears to be a transient effect of FF therapy, frequently observed and generally reversible upon discontinuation of the medication [34]. Taken together, careful monitoring of serum biomarkers may be warranted in older patients receiving fenofibrate.

We found that the old as compared to young rats’ myocardium showed some disorganization of the heart microarchitecture, a common observation in rodents [35]. Another frequent finding in the aging heart is fibrosis, which may contribute to cardiac diseases [36]. Interestingly, although the collagen-stained area was similar in left ventricles of control rats in both age groups, the higher FF dose clearly increased fibrosis in the old heart. Hayashi et al. [37] reported highly heterogeneous fibrosis levels in the old rats’ hearts, whereas Lin et al. [38] noted that in C57BL/6J senescent mice interstitial fibrosis was punctate, indicating reparative processes. Although there is a general consensus that aging-associated interstitial and perivascular fibrosis results in myocardial muscle stiffening and altered heart function, the differences in species, gender, rodent strains, and methods of fibrosis assessment may account for the discrepancies between the results of various studies [39,40,41]. Nevertheless, our finding that FF exacerbated fibrosis in the old rat heart should be treated as a warning against using high doses of fibrates in the elderly.

We suggest that the mechanism explaining the exacerbation of myocardial fibrosis by high-dose FF in old rats could involve oxidative stress. In rats treated with very high doses of PPARα agonists, cardiac structural damage was linked to marked stimulation of FAO resulting in a metabolic overload [32]. These conditions could be aggravated in the aged hearts, which display reduced FAO and diminished metabolic flexibility [42]. In this setting, FF-driven FAO acceleration could lead to increased reactive oxygen species generation and oxidative stress that may activate profibrotic pathways (e.g., TGF-β/SMAD), increasing collagen deposition. Notably, studies in younger or disease-model hearts report antifibrotic action of FF by increased matrix metalloproteinase activity [43] or through enhancement of autophagy [44]. Thus, age–drug interactions may shift fenofibrate’s usual profile of action toward injury.

Immunohistochemical staining for markers of cardiac tissue remodeling revealed substantially higher VEGFB immunoreactivity in control old than in young rats, with no effect of either dose of FF. This novel observation suggests that increased VEGFB expression could present a beneficial adaptive response to the aging-associated heart’s fibrosis. It has been recently documented by cardiomyocyte-specific gene transfer or knockout that, apart from the well-known role of VEGFB in physiological angiogenesis and adaptive cardiac muscle hypertrophy, this growth factor plays a role in reprogramming myocardial metabolism [45,46,47], e.g., in old mice, heart-specific overexpression of VEGFB induced compensatory, cardioprotective hypertrophy [46] and improved insulin sensitivity through downregulation of cardiac lipoprotein lipase (LPL) activity [47].

In contrast to VEGFB, NPPA immunoreactivity in the old rats’ myocardium remained comparable to that of young controls. Contrary to our results, Yu et al. found increased *Nppa* mRNA levels in the hearts of 20-month-old versus 8-week-old mice [48]—a discrepancy that warrants further studies. The *Nppa* gene encodes atrial natriuretic peptide (ANP), which promotes natriuresis, vasodilation, and inhibition of the renin–angiotensin–aldosterone axis, thereby reducing cardiac preload and blood pressure [49]. After birth, NPPA expression is restricted to cardiomyocytes [50], primarily of the atria. However, under pathological conditions such as cardiac hypertrophy or heart failure, NPPA expression is reactivated in ventricular cardiomyocytes, serving as a biomarker of myocardial stress [51,52]. A novel finding of our study is that in both young and old rats FF increased NPPA expression, which may suggest a reactive anti-hypertrophic effect of the drug.

We are probably the first to describe cardiac effects of fenofibrate in old rats, since almost all studies that characterized FF action on the mammalian heart were performed in young animals, mainly rats and mice. Generally, research in various experimental models leading directly or indirectly to the heart’s insufficiency documented preventive and cytoprotective effects of fibrates [13,43,44,53], e.g., in abdominal artery constriction-induced hypertensive Sprague-Dawley rats, treatment with FF reduced cardiac hypertrophy and remodeling [54]. Likewise, two-month-long FF treatment of seven-week-old Wistar-Kyoto rats subjected to renovascular hypertension counteracted the development of hypertension and the increase in left ventricular mass, and reversed the Ang II-induced upregulation of *Nppa* mRNA expression in cultured cardiomyocytes [55]. Thus, even though FF was shown to protect against cardiac hypertrophy in various pathological models of heart function depression in young rodents [14,53,55], we found that FF does not prevent structural alterations associated with physiological aging in the rat heart, and may even exacerbate deposition of collagenous tissue when administered at a high dose.

Detection of intracellular lipid depots in cryosections by Oil Red O staining has been recently established as a semiquantitative method of lipids’ measurement in cells, in contrast to biochemical techniques of lipid extraction. Unexpectedly, we observed increased accumulation of lipid droplets in the cardiomyocytes of both young and old rats treated with high-dose FF. Although this finding may seem paradoxical in view of the drug’s agonism to PPARα, we suggest that this may result from dose-related metabolic overload, not seen in earlier studies using moderate FF dosing in non-aged animals. FF has been shown to increase the expression of fatty acid transporter proteins in multiple cell types [56], and, with incomplete efficiency of FAO, such an increased lipid uptake could result in increased lipid deposition in cardiomyocytes. In support, chronic activation of PPARα resulted in cardiomyocyte lipid accumulation and cardiac dysfunction in PPARα-transgenic mice, which could be reversed through downregulation of the fatty acid transporter CD36 [57]. Our morphological findings confirm the biochemical study of Koonen et al., who demonstrated increased levels of myocardial triacylglycerol and fatty acids in the hearts of 52-week-old as compared to 10-week-old mice, which was attributed to a combination of increased lipid influx due to upregulation of CD36 and impaired FAO [58]. Consistently, our gene expression analyses showed that selected genes involved in the transfer of long-chain fatty acids into mitochondria (i.e., *Cpt1*, carnitine palmitoyltransferase 1) and their mitochondrial oxidation (i.e., *Lcad*, acyl-CoA dehydrogenase, long chain) were indeed downregulated in the old hearts. Yet, aging did not affect the mRNA levels of *Acox1* and *Mcad* (acyl-CoA oxidase 1 and acyl-CoA dehydrogenase, medium chain, respectively), whose protein products participate in peroxisomal and mitochondrial oxidation of fatty acids, indicating that aging may selectively downregulate only certain components of the FAO pathway. Mechanistically, a decrease in the cardiac fatty acid utilization has been implicated in lipotoxic cardiomyopathy as a contributor to cardiac function decline in the elderly [58].

We also showed that, depending on age, FF elicits different effects on the cardiac expression of molecular targets involved in energy metabolism. The heart of control old rats showed decreased expression of PGC-1α, a central regulator of mitochondrial biogenesis and metabolic adaptation, independently of the protein levels of the key metabolic sensors, PPARα and AMPK, which were unaffected by aging. Our results corroborate previous studies, which demonstrated that an age-related PGC-1α decline may contribute to reduced mitochondrial capacity and compromised oxidative phosphorylation [59,60,61].

As a PPARα agonist, FF may stimulate AMPK activity and lipid catabolism through mitochondrial and peroxisomal oxidation of fatty acids [62]. In our non-pathological setting, however, FF did not affect the active phospho-AMPK content in the young rats’ heart, in contrast to an increase in old rats treated with high-dose FF. Similarly, FF elevated PGC-1α protein content only in the aged heart, more significantly after low-dose than a trend after high-dose. These observations contrast with the outcomes of FF treatment in pathological settings, e.g., phospho-AMPK upregulation in hypertensive young rats [55] or increased protein levels of PPARα and PGC-1α in rabbits with atrial fibrillation [63]. Interestingly, no changes in PPARα and PGC-1α expression were noted in the sham-operated rabbits treated with FF [63], while there was downregulation of cardiac PGC-1α in young mice administered 100 mg/kg/day FF for 14 days [64].

Although in our study PPARα protein levels in the heart did not change upon treatment with FF, upregulation of PPARα transcriptional targets *Acox1* and a trend for *Cpt1* suggest mild activation of this receptor activity, similarly to previously published data [63,65]. On the other hand, FF did not change *Mcad* and *Lcad* expression, thus showing limited, selective, and tissue-specific effects of FF. In the same model, we have previously reported FF induction of *Acox1*, *Cpt1*, and *Lcad* in the liver of young and old rats [66], but minimal metabolic effects in the kidney [23]. Thus, the observed mild activation of FAO-related gene expression by FF under conditions of physiological aging suggests a limited metabolic response in the absence of overt stressors. In contrast, cardioprotective effects of FF reported for young animals in pathological models of hypertension [13,43,44,53,54,55], myocardial ischemia followed by reperfusion injury [67,68], and diabetes [25,69] have been attributed in part to its ability to enhance mitochondrial function and promote FAO through PPARα activation. Notably, under adverse circumstances, the heart typically undergoes a metabolic shift from FAO to glucose utilization [70,71]. Our findings, however, do not support FF repurposing to boost age-related cardiac dysfunction under non-pathological conditions.

Sterol regulatory element binding proteins (SREBPs) belong to a family of transcription factors that control the expression of enzymes required for intracellular synthesis of key lipids, such as cholesterol, fatty acids, triglycerides, and phospholipids. The SREBP-2 isoform, encoded by the *Srebf2* gene, plays a key role in the regulation of cholesterol homeostasis by activating the transcription of 3-hydroxy-3-methylglutaryl coenzyme A reductase (HMG-CoA reductase), the key enzyme of the cholesterol biosynthesis pathway [72,73]. SREBP-2 has been investigated mainly in the liver, intestine, and adipose tissue under various conditions [73]; however, we are not aware of research that targeted its expression in the myocardium of young and old rats. Aging had no effect on cardiac mRNA levels of *Srebf2*, suggesting that transcriptional regulation of the cholesterol synthesis pathway might be stable in ageing. Interestingly, we found that at a high dose, FF increased *Srebf2* expression in the myocardium of young but not old rats. Strategies designed to improve cardiac lipid metabolism should be considered for treating an age-related decline in the heart’s mechanical function.

Although under normal conditions fatty acid oxidation rather than glucose utilization secures the heart’s requirement for energy, under stressful circumstances, like hypoxia or excessive overload, glucose metabolism becomes an important source of ATP in the heart [70,71]. At insufficient oxygen delivery, lactate, and not pyruvate, becomes the final by-product of the glycolysis pathway [74]. Phosphofructokinase (PFK), a rate-limiting glycolysis enzyme, catalyzes synthesis from fructose 6-phosphate of either fructose 1,6-bisphosphate (by PFK1 isoform) or fructose 2,6-bisphosphate (by PFK2) [75]. The tetrameric structure of PFK consists of various proportions of the three isoforms typical for striated muscles, liver, and platelets (PFKM, PFKL, and PFKC or-P subunits, respectively), with the heart expressing mainly the PFKM type [75]. Mhaskar and Dunaway found that the activity of PFKM in the left ventricle of 12-month-old and 24-month-old rats was 40% and 30% lower than in 3-month-old animals, respectively [76]. We found similar *Pfkm* mRNA levels in young and old rats; yet the activity of PFK enzymes is mainly dependent on the levels of their allosteric activators/inhibitors and other modifiers [74,75]. Our finding that, under normal conditions, aging did not affect PFKM protein content but low-dose FF decreased it in the heart of old rats, adds new data to the possibility of negative effects of fibrates on the heart’s energy production under stress conditions.

Another noteworthy finding of our study is that aging did not affect the mRNA and protein levels of two sirtuins, SIRT1 and SIRT3, in the rat heart. Taking into account the important roles of SIRT1 and SIRT3 as sensors and regulators of cells’ fuel and energy metabolism [77,78], this observation suggests that under normal conditions the aged myocardium may efficiently support the heart’s function. These results stand in contrast to the findings of decreased SIRT1 levels in the hearts of 20-month Sprague-Dawley rats [79] and in 24–26-month mice [80] as compared to their young counterparts. Likewise, SIRT3 protein levels were lower in the myocardia of aged compared with young mice [80,81]. Moreover, both sirtuins’ levels were lower in the myocardium of old mice than in young animals, and acute myocardial ischemia followed by reperfusion injury led to further decreases in the sirtuins levels in both age groups, accompanied by mitochondrial relocation of SIRT1 that resulted in SIRT3-related protein mobilization [82]. The divergence between our and other reports on SIRT1 and SIRT3 in the aging heart warrants further studies.

Treatment with FF did not change SIRT1 or SIRT3 cardiac expression in young and old rats. In the same model, we have previously shown that SIRT1 expression in the liver decreased after either dose of FF in both young and old rats [66], while in the kidney, SIRT1 expression increased only after the higher FF dose and only in young rats [23]. In the hearts of mice with streptozotocin-induced diabetes [44], as well as in rabbits with electrically induced atrial fibrillation [63], FF increased SIRT1 levels compared to model groups, but not above those of the sham-operated animals [63]. With regard to SIRT3, published data indicate that FF may indirectly stimulate SIRT3 activity through its effects on SIRT1, enhancing overall cardiac resilience [83]. These results show that the effects of FF on SIRT1 and SIRT3 expression are highly dependent on the tissue/organ and the physiological/pathological context and age.

A key limitation of our study is the absence of direct assessment of cardiac function, e.g., by echocardiography. Without correlating our data to indices of cardiac performance, we cannot conclude whether the FF-induced morphological and molecular changes translate into clinically relevant impairment. Likewise, measurement of specific biomarkers of cardiac injury, such as cardiac troponins and CK-MB (creatine kinase–myocardial band), would be more informative than the serum markers of muscle tissue integrity assessed in our study. What is more, assays for metabolic and enzymatic activities could shed more light on the results concerning FF-related molecular response in the aging heart. Lastly, only male rats were included in the experiment, so sex-related differences could not be examined. Future studies should address these limitations to provide a more comprehensive understanding of fenofibrate’s effects on the aging heart.

## 4. Materials and Methods

### 4.1. Animals and Experimental Procedures

The study involved male Wistar-Han rats, bred under standard laboratory conditions at the Academic Animal Experimental Centre in Gdańsk, Poland. All experimental procedures on the animals were approved by the Local Ethical Committee in Bydgoszcz (protocols 41/2017, 58/2017, 40/2018, and 5/2019) and conducted according to institutional and European guidelines for animal experimentation.

Young adult (4-month-old) and old (24-month-old) rats were divided into treatment and control groups. Animals fed standard rodent chow (Labofeed H, Wytwornia Pasz Morawski, Kcynia, Poland) served as the control groups. Other rats were administered FF for 30 days, at 0.1% or 0.5% dose (*w*/*w*), mixed into the chow at the stage of pelleting (*n* = 8–10 animals in each young and old group; FF sourced from Glentham Life Sciences, Corsham, UK). The 30-day treatment period was established based on prior cardiovascular studies evaluating PPARα agonists in rodents, since this timeframe captures drug-driven cardiac remodeling [84] while minimizing confounding from longer-term systemic adaptations and age-related attrition.

The initially tested 0.5% FF dose, equivalent to around 100 mg/day based on the pre-experiment food intake, corresponded to approximately 260 mg/kg in young rats and 210 mg/kg in older rats. Due to mild hepatotoxicity observed at this dose [21], a separate experiment tested the 0.1% FF dose, which corresponded to approximately 52 mg/kg in young rats and 42 mg/kg in the older ones. The results for the control groups in both experiments were combined since it was found that they did not differ statistically.

Throughout the 30-day treatment period, food consumption was monitored every other day by weighing the leftover chow from pre-measured samples. Body weights were monitored twice weekly. At the end of the treatment, the rats were fasted overnight, then euthanized under full anesthesia (5% isoflurane) and blood was collected via cardiac puncture. Samples of the heart muscle, including the apex and fragments of the left ventricle, were quickly collected and flash-frozen in liquid nitrogen for molecular analyses, or preserved in 4% paraformaldehyde for histological assessment.

### 4.2. Serum Levels of Lipids and Biochemical Markers

Measurements of triglycerides, total cholesterol, creatine kinase (CK), lactate dehydrogenase (LDH), and creatinine levels in fresh blood sera were performed on the same day with routine diagnostic tests by the Veterinary Diagnostic Laboratory Lab-Wet (LabWet, Gdańsk, Poland). The data on triglycerides, total cholesterol, and creatinine levels were previously published in [21,23] and are reused here with permission to provide context for the present analyses.

### 4.3. Histological Stains

Formaldehyde-fixed samples of the heart (mid-level transverse section) were processed and embedded in paraffin using a tissue processor (STP 120, Zeiss, Jena, Germany), then sectioned (4 μm thickness) using a rotary microtome (Opta-Tech MR-315, Warsaw, Poland) and mounted on glass slides (Super-Frost Ultra Plus, Thermo Scientific, Vantaa, Finland). The sections were stained following standard protocols [85]: with hematoxylin and eosin (HE; topological examination) and Mallory’s trichrome method (for collagenous tissue area assessment).

Evaluation of lipid droplets’ content in the heart was performed by Oil Red O staining on 5 μm-thick cryosections of the heart (Leica CM1950, Kawaska, Warsaw, Poland). The sections were post-fixed in 10% formal calcium for 45 min, then washed three times in distilled H_2_O (dH_2_O) and briefly in 60% triethyl phosphate, followed by staining with Oil Red O (Carl Roth GmbH + Co. KG, Karlsruhe, Germany) solution in triethyl phosphate for 20 min. After washing in dH_2_O, the slides were stained with Harris’s hematoxylin solution (Sigma-Aldrich), followed by blueing in a saturated lithium carbonate solution, washing in dH_2_O, and embedding in glycerol jelly.

Microscopic examination of the slides was carried out using an Olympus Cell-Vivo IX 83 microscope (Olympus Corp., Tokyo, Japan) equipped with a digital camera (SC-50, Olympus Corp.), and microphotographs were captured at 10× and 20× magnifications. Image analyses were performed with the cellSens Dimension 4.1 software (Olympus Corp.): a neural network was designed for quantification of the collagenous tissue area fraction (stained by Mallory’s trichrome method), while quantification of lipid droplets’ area fraction (Oil Red O-stained) was achieved with the Count and Measure function on RGB-separated images with appropriate threshold setup. For each type of staining, analyses were carried out for 4–6 rats per group, in 1–2 non-contiguous sections, and at least six regions of interest (ROIs) per slide.

### 4.4. Immunohistochemical Analyses

Immunohistochemical (IHC) staining of heart muscle sections was conducted following the method described by Kieżun et al. (2022), with some modifications [86]. Antigen retrieval was performed by microwaving the sections for 6 min in Retrieval Solution (Leica Microsystems, Wetzlar, Germany) at pH 6.0, followed by treatment with 3% H_2_O_2_ in methanol for 10 min to inhibit endogenous peroxidase activity. To block nonspecific binding sites, the sections were incubated with 2.5% normal horse serum (Vector Laboratories, Birmingham, CA, USA) for 30 min. Subsequently, the sections were incubated overnight at 4 °C with rabbit polyclonal antibodies against VEGFB (#A12689, ABclonal, Woburn, MA, USA) diluted 1:200 in phosphate-buffered saline (PBS), or NPPA (#27426-1-AP, Proteintech, Rosemont, IL, USA) diluted 1:400 in PBS. Secondary antibody incubation was carried out using the ImmPRESS Universal reagent Anti-Rabbit Ig (Vector Laboratories, Birmingham, CA, USA) for 30 min. The specificity of the immunostaining was confirmed by omitting the primary antibody and replacing it with rabbit serum. Visualization of the sections was performed using the Liquid DAB + Substrate Chromogen System (Dako, Carpinteria, CA, USA), followed by counterstaining with hematoxylin (Sigma-Aldrich, Merck KGaA, St. Louis, MO, USA), dehydration through a graded ethanol series, clearing in xylene, and mounting in DPX (Sigma-Aldrich).

The labeled tissue sections were photographed using an SC-50 camera (Olympus Corp.) attached to an Olympus Cell-Vivo IX 83 microscope (Olympus Corp.). For each target antigen, measurements of the IHC-positive areas were performed using the Count and Measure function of the cellSens Dimension 4.1 software (Olympus Corp.) for RGB-separated images with appropriate threshold setup, for 4–6 rats per group, in at least six ROIs per slide.

### 4.5. Reverse Transcription and Quantitative Real-Time PCR

RNA, extracted from homogenized samples of the heart muscle using the Total RNA Mini Plus kit (A&A Biotechnology, Gdynia, Poland), was reverse-transcribed with the smART First Strand cDNA Synthesis kit (EURx, Gdańsk, Poland). qPCR reactions were carried out with 5× diluted cDNA samples (6–9 per group), SG (SybrGreen) qPCR Master Mix (EURx), and 0.25 µM of sense and antisense primers (Sigma-Aldrich, Merck KGaA, St. Louis, MO, USA). Primer sequences were designed with Primer-BLAST (NCBI, National Center for Biotechnology Information, Bethesda, MD, USA), and are listed in Supplementary Table S1. All PCR reactions were followed by dynamic melting curve analysis to ensure specificity of the amplification. Gene expression levels were analyzed using the 2^−ΔΔCT^ method. The internal controls for normalization of gene expression were *Arbp* and *Rplp1* (geometric mean), chosen from among five housekeeping genes using the comparative delta-CT method [87].

### 4.6. Western Blotting for Protein Quantification

Whole-cell lysates (4–6 per group) were prepared with Mammalian Cell Extraction Kit (BioVision, Miliptas, CA, USA), with the addition of protease and phosphatase inhibitors (#P8340, Sigma-Aldrich, Merck KGaA, St. Louis, MO, USA, and #78420, Thermo Scientific, Vantaa, Finland, respectively). Equal amounts of protein samples (~20 μg) were separated on 10% SDS-PAGE gels and transferred to PVDF membranes (Bio-Rad, Hercules, CA, USA). After blocking for 1 h in 5% skimmed milk in TBS-T, the membranes were incubated overnight at 4 °C with primary antibodies. The antibodies were MCAD (#67742-1-Ig, Proteintech, Rosemont, IL, USA), PFKM (#55028-1-AP, Proteintech), PGC1α (#bs-7535R, Bioss), PhosphoPlus^®^ AMPKα (Thr172) antibody duet (#8208S, Cell Signaling Technology, Leiden, The Netherlands), PPARα (#66826-1-Ig, Proteintech), SIRT1 (#bs-0921R, Bioss), and SIRT3 (#5490, Cell Signaling Technology). The levels of GAPDH (#AB2302, Sigma-Aldrich, Merck KGaA, St. Louis, MO, USA) served as the loading control. After washing, the membranes were incubated for 1 h with appropriate AlexaFluor^®^-conjugated secondary antibodies (anti-rabbit or anti-mouse, respectively: #111-625-144, #715-655-150, Jackson ImmunoResearch, West Grove, PA, USA). Detection of the protein bands was carried out with Odyssey^®^ CLx imaging system (LI-COR^®^ Biosciences, NE, USA) and quantified by densitometric analysis using the ImageQuant™ TL 10.0 software (Cytiva Europe GmbH, Freiburg im Breisgau, Germany).

### 4.7. Statistical Analyses

Statistical analyses were performed using GraphPad Prism v.6 (GraphPad Software, San Diego, CA, USA). After checking that the datasets came from a normally distributed population (Shapiro–Wilk test), statistical analyses were performed using one-way ANOVA with Dunn’s multiple comparisons test, or unpaired Student’s *t*-test for two-group comparisons, following the ROUT test for outliers. Data are presented as mean ± standard error (SEM). Statistical significance was set at *p* < 0.05. Differences between groups are denoted with asterisks: * *p* < 0.05, ** *p* < 0.01, *** *p* < 0.001.

## 5. Conclusions

We studied the cardiac effects of the lipid-lowering drug fenofibrate during physiological aging, which in rat is commonly associated with mild hyperlipidemia. The purpose of choosing this rat model was to isolate age as the primary modifier of the drug effect, although in a setting justifying the use of lipid-lowering therapy. Our findings suggest that the cardiac muscle response to fenofibrate is influenced by age and dosage. In particular, in the aged heart, high-dose FF may exacerbate myocardial fibrosis while enhancing the expression of PGC-1α and phospho-AMPK, and, more significantly than in the young heart, stimulate intracellular lipid accumulation and cardiac hypertrophy markers. Our results raise concerns about fenofibrate’s effects on cardiac health in older age, although further studies evaluating cardiac performance are needed. Based on our rat data, we hypothesize that elderly patients without advanced dyslipidemia or cardiac pathology may be at risk of adverse myocardial remodeling with fenofibrate treatment due to aging-related metabolic inflexibility of the cardiac muscle. Our findings, therefore, do not support the use of fenofibrate for primary prevention scenarios. Moreover, our results underscore the importance of considering dose optimization and age-dependent responsiveness to fibrates, the important class of lipid-lowering drugs, when designing therapeutic strategies in the elderly.

## Figures and Tables

**Table 1:** Blood serum levels of triglycerides (TG), total cholesterol (Chol), lactate dehydrogenase (LDH), creatine kinase (CK), and creatinine in young and old rats treated with 0.1% or 0.5% fenofibrate (FF). The data are means ± SEM, 7–20 animals per group. *, *p* < 0.05, **, *p* < 0.01, ***, *p* < 0.001, vs. control animals of the same age; #, *p* < 0.05, ###, *p* < 0.001, in old vs. young control rats. The data on triglycerides and total cholesterol were previously published in [21], and creatinine levels were previously reported in [23]. These data are reused here to provide context for the present analyses.

	Control	0.1% FF	0.5% FF	Control	0.1% FF	0.5% FF
	Young Rats			Old Rats		
TG	53.18 ± 5.50	40.44 ± 5.13	46.50 ± 4.18	114.5 ± 10.50 ^###^	91.91 ± 9.66	52.11 ± 5.57 *
Chol	66.24 ± 4.265	26.78 ± 2.697 *	44.00 ± 4.856	110.2 ± 5.754 ^#^	78.10 ± 3.247	53.67 ± 11.84 **
LDH	795.1 ± 94.12	672.3 ± 110.2	922 ± 128.6	969.8 ± 130.5	1052 ± 153.4	920.2 ± 193
CK	1392 ± 163.5	1132 ± 320.8	3179 ± 1104	1113 ± 211	1416 ± 219.1	863.8 ± 267.6
Creatinine	0.67 ± 0.02	0.63 ± 0.02	0.77 ± 0.04 *	0.74 ± 0.03	0.77 ± 0.06	0.99 ± 0.04 ***

## Data Availability

The original contributions presented in this study are included in the article. Further inquiries can be directed to the corresponding author.

## Supplementary Materials

The following supporting information can be downloaded at: https://www.mdpi.com/article/10.3390/ijms26168038/s1.
