# Supplementary material for: Fenofibrate Differently Affects the Heart’s Morphology and Metabolism in Young and Old Rats
Source: Int J Mol Sci. 2025 Aug 20;26(16):8038. doi: 10.3390/ijms26168038 (PMC12386944; doi:10.3390/ijms26168038)
Supplement: Supplementary file 1 [file ijms-26-08038-s001.zip › Supplementary Table S1 List of primers.pdf]

Supplementary Table S1: List of primers.

Gene symbol	Gene name	Forward and reverse primer sequences
Acox1	Peroxisomal acyl-coenzyme A oxidase 1	GTCTCTTGTATTCTCTCTATGG<br>GTAAGATTCATGGACCTCTG
Arbp0	60S acidic ribosomal protein P0<br>*(housekeeping gene)*	CTCAGTGCCTCACTCCATCA<br>GGGGCTTAGTCGAAGAGACC
Cpt1	Carnitine palmitoyltransferase 1	ATGTTTGACCCAAAGCAGTACCCC<br>TCGCCTGCGATCATGTAGGAAAC
Lcad (Acadl)	Long-chain acyl-CoA dehydrogenase	TCGAGCAGTTTATCCCCCAG<br>TGAACACCTTGCTTCCATTGAG
Mcad (Acadm)	Medium chain acyl-CoA dehydrogenase	GCAGCTGATGATGTGTGCCTA<br>ATCTGGGTTAGATCGCGTCA
Pfkm	Phosphofructokinase, muscle type	TTTCCCCAAGGACAATCTGC<br>AGAGGTCAACACGGCGA
Rplp1	Ribosomal protein lateral stalk subunit P1<br>*(housekeeping gene)*	AAGCAGCTGGTGTCAATGTTG<br>CAGCCCCTACATTGCAGATGA
Sirt1	Sirtuin 1	CAGAACCACCAAAGCGGAAAAA<br>GAAACCCCAGCTCCAGTCAGAA
Sirt3	Sirtuin 3	AAGCTGGTTGAAGCTCATGGGTC<br>TCCAGGGAGGTCCCAAGAATGAG
Srebf2	Sterol regulatory element-binding transcription factor 2	AGCAGTCTCAATGTCAGCGG<br>TGCCAGAGTGTTGTCCTCAG
